# Does a microbial-endocrine interplay shape love-associated emotions in humans? A hypothesis

**DOI:** 10.1128/msystems.00415-25

**Published:** 2025-07-14

**Authors:** Jake M. Robinson, Ondi L. Crino, Araceli Camargo, Martin F. Breed

**Affiliations:** 1 College of Science and Engineering, Flinders University 117627, Bedford Park, South Australia, Australia; 2 The Aerobiome Innovation and Research Hub, Flinders University 1065, Bedford Park, South Australia, Australia; 3 Centric Lab, London, United Kingdom; University of California San Diego, La Jolla, California, USA

**Keywords:** endocrinology, love, microbiome, microbiota-gut-brain axis

## Abstract

Emerging evidence suggests that the human gut microbiome plays a regulatory role in the endocrine system, influencing hormone activity and potentially shaping the emotional and physiological dimensions of behaviors such as aggression and emotional states, including love. Here, we review the hypothesis that microbial-endocrine interactions shape love-associated emotions by modulating hormone levels and activity. While the division of love into discrete components such as lust, attraction, and attachment is somewhat artificial and may not fully capture the complexity of human experience, the associated emotions nevertheless correspond to well-characterized neuroendocrine processes. Psycho-endocrinological theory proposes that the complex and nonlinear interplay between testosterone, estradiol, and other hormones drives emotions associated with lust; serotonin and dopamine play a role in emotions linked to attraction; and oxytocin and vasopressin influence the modulation of attachment and social bonds. Microbial communities may influence emotions and relationship dynamics through effects on the hormonal pathways linked to love-associated emotions. While non-human animal studies suggest microbiota can impact social bonding, sexual preferences, and stress-related behaviors, direct evidence in humans remains limited. Investigating the evolutionary underpinnings of microbial-endocrine interactions could provide insights into how microbes influence emotions beyond love, including hate and aggression—with important societal implications. Indeed, understanding these mechanisms could have profound implications for human relationships, mental health, and even therapeutic interventions targeting the microbiota-gut-brain axis. Future research is needed to unravel the extent to which microbiomes contribute to the neurobiology of love (and other emotions) and whether manipulating microbial communities could modulate emotional and social behaviors.

**IMPORTANCE:**

Love is often considered an abstract emotion, but emerging science suggests that it may be shaped by the microscopic inhabitants of our bodies: microbes. This paper explores the intriguing hypothesis that microbes can influence the hormonal and neural systems linked to love-associated emotions—via the microbiota-gut-brain axis. Drawing on animal studies and early human microbiome and endocrine research, we highlight how microbes modulate neurohormones like oxytocin, dopamine, and testosterone, which play key roles in social bonding. By regulating these systems, microbes may also shape emotional and behavioral responses. This research opens new avenues for understanding not just the (micro)biology of love but also the potential for microbiome-targeted therapies to support relational well-being. By linking microbiome and emotion science, the article raises the important question of whether love is a phenomenon influenced by our resident symbionts, adding an intriguing and potentially impactful dimension to our understanding of human connection and behavior.

## INTRODUCTION

Growing evidence suggests that various aspects of human behavior and emotions are influenced by the trillions of microbial symbionts within our bodies (1). Indeed, the human gut microbiome (the consortium of microorganisms, including bacteria, fungi, viruses, and archaea, and their theatre of activity) can affect various aspects of our behaviors, cognition, and attractions, including decision-making (2), emotional regulation (3), desires to exercise (4), sexual preferences (5), and cognition (6). Several pathways link microbiota (particularly bacteria and their metabolites) to these host neuropsychological traits, including the microbiota-gut-brain axis. For example, a host’s gut microbiota can influence neurological processes via secreting metabolites and stimulating the vagus nerve—the largest cranial nerve linking the brain to several peripheral organs (1). Moreover, host microbiota can affect the synthesis and/or regulation of hormones and neurotransmitters, including dopamine, serotonin, and estrogens (7). In this way, it might be possible for our gut microbiomes to influence love-associated emotions (and other emotions such as aggression) through effects on endocrine function.

Love is a complex emotion that is immensely significant to the human experience. Indeed, in acknowledgment of its complexity, it is essential to recognize the multifaceted dimensions of human existence, including the diversity of gender identities and sexual orientations. We also acknowledge that multiple levels of biological sex can be considered (e.g., hormonal, gonadal, and chromosomal). Here, we discuss endocrine patterns (sex steroid synthesis and secretion) typical of cisgender males and females (i.e., XY and XX individuals). However, we recognize that an individual’s gender can be independent of their chromosomal and endocrine profiles and that endocrine profiles can vary independently of chromosomal identity (e.g., for XY individuals with three-beta-hydroxysteroid dehydrogenase deficiency). Human relationships and the phenomenon of love are intricate, deeply personal experiences that defy adherence to a standard narrative. Nonetheless, from a biological perspective and despite the complexity, the diverse human love-associated behaviors are influenced by a suite of hormones.

Psychologists have made generalizations about love-associated emotions and distilled them into three categories: lust, attraction, and attachment (8, 9). We will use this tripartite framework as a heuristic device to explore the potential biological underpinnings of love-associated emotions, while acknowledging that these categories are somewhat artificial and overlapping. Accordingly, our language refers to emotions associated with lust, attraction, and attachment, rather than treating them as discrete or universally experienced states.

Lust-associated emotions are often conveyed as the human desire for sex (although we recognize it may not be viewed the same by all individuals and in all cultures) and have been linked to an evolutionary drive to reproduce (10). It should be noted that emergent emotions not driven by evolutionary or reproductive processes may also play a role in lust-associated emotions. In comparison, attraction is viewed as an emotion-motivation system that drives exhilarating and often obsessive thoughts about another person (11). Attachment is often involved in long-term relationships and is thought to have evolved to “enable individuals to cooperate with a reproductive mate until species-specific parental duties have been completed” (11). Attachment-associated emotions could also be driven by non-reproductive emergent behaviors or processes that indirectly affect reproduction (e.g., via co-parenting models).

From a biological perspective, each broad emotion associated with such emotions is distinguished by a unique set of hormones and neurotransmitters stemming from the brain and various peripheral organs (12). Lust-associated emotions are predominantly driven by testosterone and estradiol; attraction-related emotions by dopamine, noradrenaline (norepinephrine), and serotonin (5-HT); and attachment by oxytocin and vasopressin (11, 13). Other hormones, such as corticosteroids, which indirectly influence the hypothalamic-pituitary-gonadal axis (HPG axis), also likely play a role in these emotions (14).

Overall, we have a good understanding of the hormone systems that play important roles in forming emotions relating to love. But what factors influence the secretion of hormones that, in turn, affect human love-associated emotions? As American neuroscientist Robert Sapolsky said: “any given type of explanation [for a behaviour] is the end product of the influences that preceded it” (15). Given such perspectives, we know that emotions (and microbiomes) are strongly influenced by environmental factors (e.g., diet, soundscape, and biodiversity exposure) (16, 17). We also know that our gut microbiomes influence the synthesis and/or regulation of hormones that, in turn, affect love-associated emotions—and the evidence is particularly strong for a bacterial influence. These physiological links open an intriguing pathway whereby the human microbiota could influence emergent cognitive properties (love-associated emotions) via changes in endocrine function.

In this minireview (synthesized as a conceptual/hypothesis article), we explore the hypothesis that the human gut microbiome and its metabolites interact with the endocrine system to influence love-associated emotions. We seek to address the question: is there a microbial influence or basis for love? We focus on the endocrine pathways linked to emotions, which themselves are associated with lust, attraction, and attachment (while recognizing the complexity of other factors involved, e.g., environment and culture) and discuss how a host’s symbiotic microbiota may affect love through changes in hormone production and action.

## IS LOVE ROOTED IN OUR GUTS?

### Lust-associated emotions

In humans (and potentially other species), the presence of lust-associated emotions is considered a powerful motivational force that may be favored by natural selection (18, 19); however, we also recognize that emergent behaviors (not driven by natural selection) also play a role in choosing sexual partners. Our endocrine system plays an important role in regulating sexual desire and behavior through the release and activity of sex hormones (20). The hypothalamus, a structure in the ventral brain, plays a central role in coordinating the endocrine system through its influence on the pituitary gland. For example, the hypothalamus mediates the secretion of the sex hormones testosterone and estradiol from the gonads that control sexual behavior (21). The hypothalamus synthesizes a range of hormones and controls the release of the sex hormones testosterone and estradiol from the gonads through its influence on the pituitary gland (22). Testosterone is commonly associated with male sexual desire, but it also plays a role in female sexual behavior, albeit at lower circulating levels, and can be aromatized into estrogens (23), which also contribute to sexual and emotional responses. It may increase libido and promote sexual arousal in different biological sexes (24). Estradiol, a type of estrogen predominantly considered a female sex hormone, affects sexual motivation in females, particularly around the time of ovulation when levels of estrogen peak (25). Sex hormones interact with the brain’s neural circuits involved in reward, pleasure, and arousal that influence the experience of sexual desire and facilitate the motivation for sexual behavior (26).

**Fig 1:**
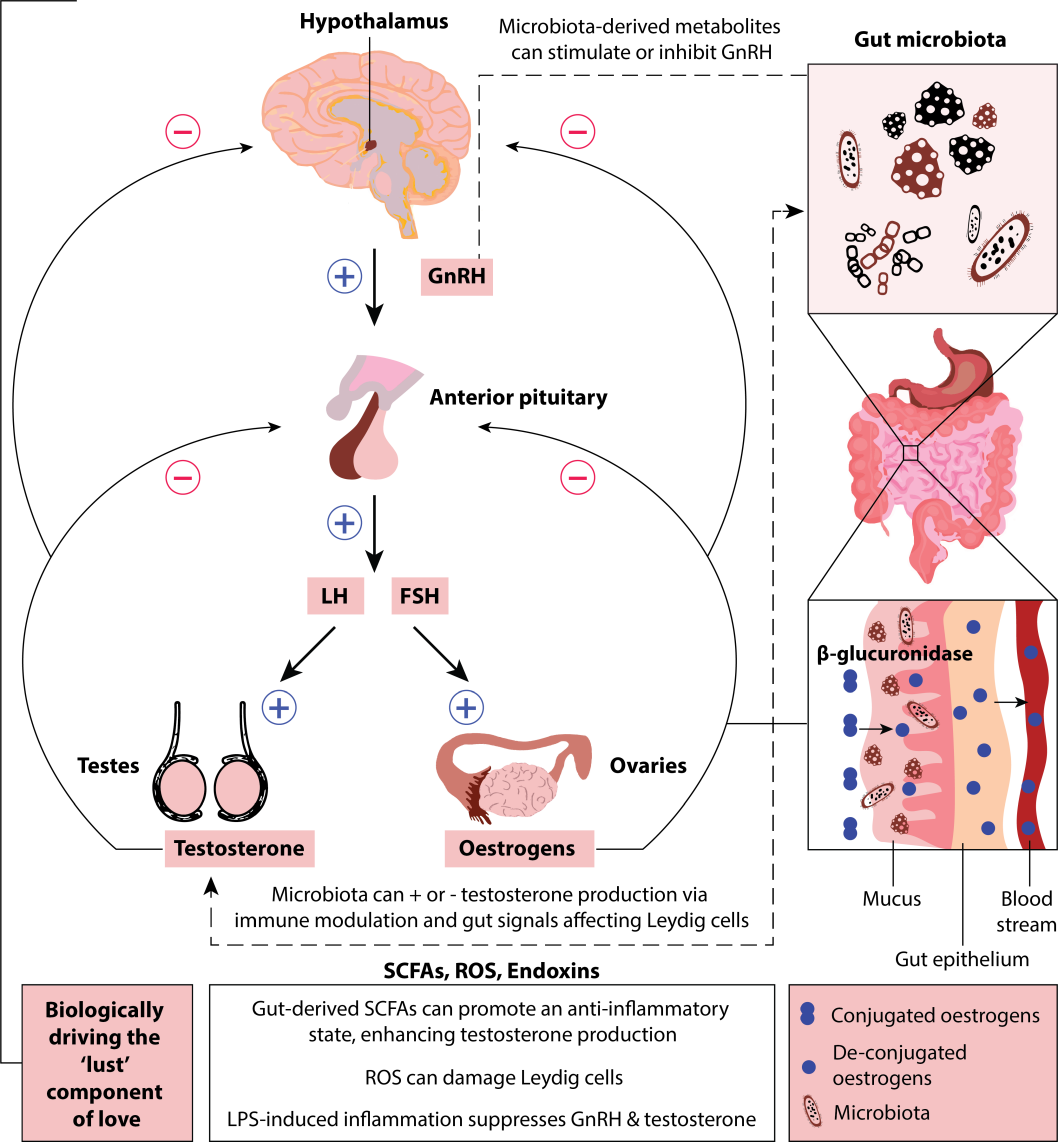
Gut microbiota can influence the synthesis and processing of sex hormones, including testosterone and estrogens (estradiol). Microbiota-derived short-chain fatty acids (SCFAs), reactive oxygen species (ROS), and endotoxins can act on Leydig cells in the testes and affect testosterone synthesis and secretion. Microbiota can also modulate levels of estrogens by producing an enzyme called β-glucuronidase, which drives deconjugation and hence levels of bioavailable estrogens.

Previous research has suggested a link between gut microbiota and the synthesis and secretion of testosterone via the HPG axis (27). The HPG axis is a neuroendocrine pathway that regulates the production and release of sex hormones by the gonads (22). As such, the HPG axis plays a crucial role in controlling reproductive functions, including the development of secondary sexual characteristics, the menstrual cycle in females, and sperm production (indirectly) in males. The HPG axis operates through negative feedback mechanisms (Fig. 1; except during the late follicular phase of the menstrual cycle). Such hormonal negative feedback is a regulatory mechanism in which rising hormone levels inhibit further hormone production to maintain homeostasis (28). When sex hormone levels are low, the hypothalamus synthesizes and releases gonadotropin-releasing hormone (GnRH), which triggers the synthesis and release of luteinizing hormone (LH) and follicle-stimulating hormone (FSH) from the anterior pituitary gland (29). Gonadotropins, in turn, stimulate Leydig cells in the testes and follicular cells in the ovaries to produce sex hormones. As sex hormone levels rise, they send signals back to the hypothalamus and pituitary gland, inhibiting the synthesis and release of GnRH and reducing LH and FSH production. An exception to this is during the late follicular phase in the menstrual cycle when high estradiol levels elicit a change from negative to positive feedback of the HPG axis, resulting in a spike in LH and FSH that stimulates ovulation. These feedback loops help regulate the production of sex steroids.

Gut microbiota can regulate androgen production and metabolism (via the release of reactive oxygen species, endotoxins, and metabolites, such as short-chain fatty acids [SCFAs]), thus influencing the HPG axis. The gut microbiome is also involved in the metabolism and intestinal deglucuronidation of dihydrotestosterone (DHT) and testosterone, resulting in extremely high levels of DHT (30, 31). DHT is thought to play a pivotal role in sexual desire and the development of reproductive organs (32). Microbially derived metabolites, including SCFAs (such as butyrate, propionate, and acetate) and secondary bile acids, may cross the intestinal epithelium via monocarboxylate transporters or passive diffusion, subsequently entering circulation and modulating endocrine signaling. Additionally, β-glucuronidase activity within the gut facilitates the deconjugation and reabsorption of testosterone and DHT through enterohepatic circulation, increasing their systemic bioavailability (Fig. 1). Endotoxins such as lipopolysaccharides (LPS) can also activate toll-like receptor 4 on immune cells, leading to inflammation-driven suppression of hypothalamic GnRH secretion, thereby indirectly modulating androgen production. These pathways highlight potential mechanisms by which gut microbiota influence hormone signaling and the physiological regulation of sexual function.

Similar to interactions between androgens and the gut microbiome, research has shown that the gut microbiome is linked to estradiol production. Estradiol affects sexual motivation in females (33) and, thus, likely has a biological influence on lust. Research has shown that the gut microbiome is a regulator of circulating estradiol (26), which has led to the identification of the estrogen-gut microbiome axis. The gut microbiome modulates estradiol levels (conjugated steroids are present in the gut because the human body has metabolized their free forms) through an enzyme called β-glucuronidase that converts estrogens into their active forms by deconjugation (34). However, when the gut microbiome is in a dysbiotic (i.e., “distressed”) state, resulting in reduced microbial diversity, the deconjugation process is hindered, leading to decreased levels of circulating estrogens (35). The estrogen-gut microbiome axis is supported by research that has shown females with hypoactive sexual desire disorder have significantly different gut microbiota and metabolic activity than females in the healthy control group (36). Overall, there is empirical support linking host microbiota, hormones, and lust- and attraction-associated emotions in humans via androgens and estrogens (Fig. 1).

### Attraction-associated emotions

Attraction-associated emotions are thought to be distinct but closely related to lust-associated emotions. While they may occur together, each can occur independently. Attraction involves neural pathways associated with “reward” behaviors, which have been invoked to explain the exhilarating and all-consuming feelings in the early stages of a relationship (34).

Dopamine is released when engaging in enjoyable activities like spending time with loved ones and having (and/or anticipating) sex (37). High levels of dopamine and noradrenaline (norepinephrine) are released during moments of attraction, primarily from the ventral tegmental area (VTA) and locus coeruleus (LC), respectively. Noradrenaline is also produced from the adrenal medulla during sympathetic nervous system activation (i.e., the “fight, flight, or freeze” response). The VTA, a key component of the brain’s reward system, projects dopaminergic neurons to the nucleus accumbens, prefrontal cortex, and amygdala, reinforcing feelings of pleasure, motivation, and reward. Simultaneously, noradrenaline from the LC enhances arousal, alertness, and focus, contributing to feelings of giddiness and euphoria and affecting energy levels and sleep behavior (8). The heightened arousal experienced during attraction might be related to noradrenaline’s role in the so-called “fight, flight, or freeze” response, which maintains alertness during stressful or potentially life-threatening activities (e.g., escaping from predators) (38).

Attraction activates the brain’s dopaminergic reward centers, such as the VTA and caudate nucleus (39). The medial orbitofrontal cortex has been implicated in rewarding experiences, including love and attraction. Takahashi et al. studied the medial orbitofrontal cortex in relation to attraction (37). They found a positive correlation between excitement levels and dopaminergic activation when subjects were exposed to a love treatment but not when exposed to control conditions.

**Fig 2:**
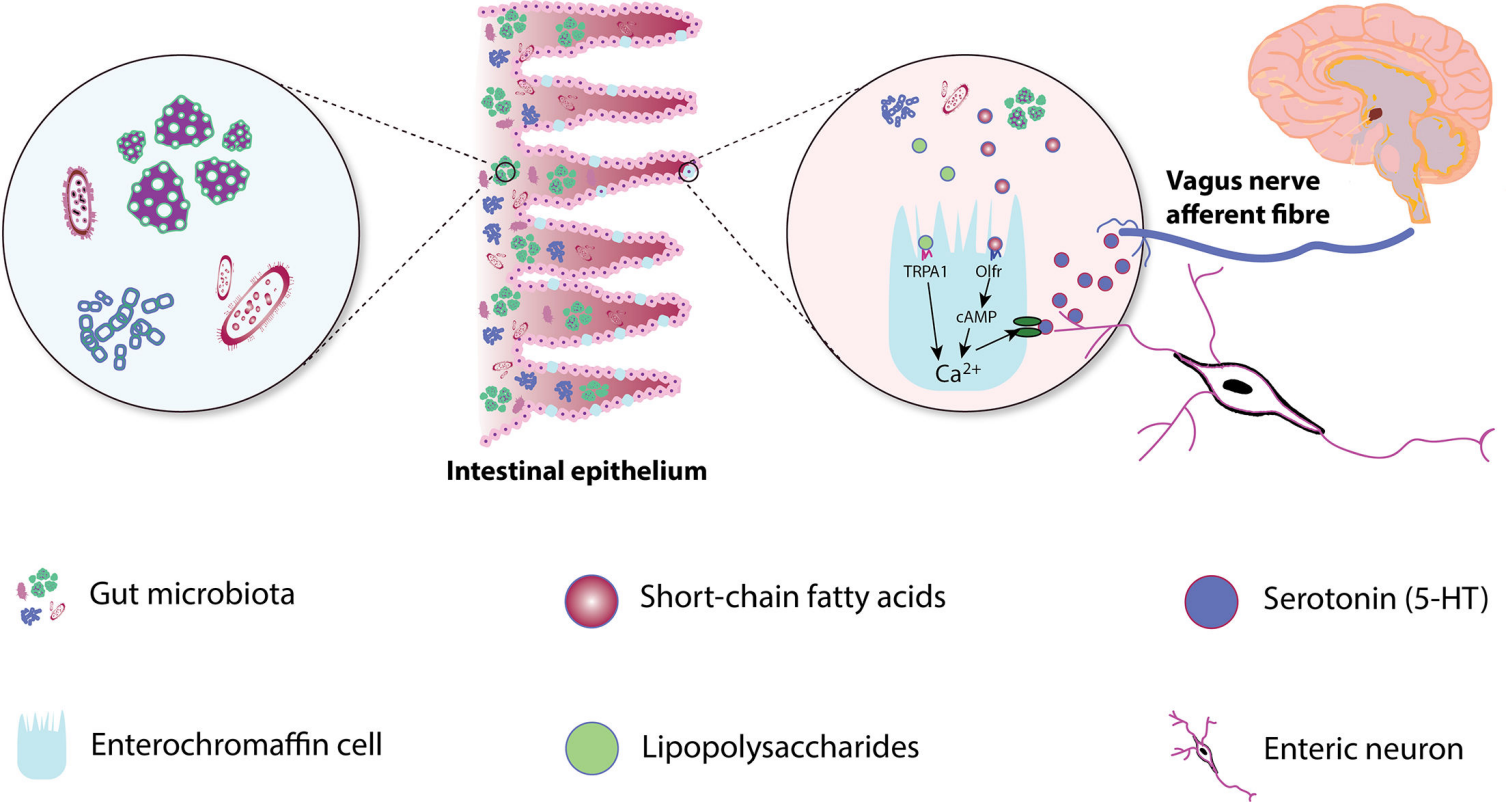
Serotonin is produced by enterochromaffin cells in the gut. Its production is stimulated by gut microbiota and their by-products, such as short-chain fatty acids (SCFAs) and lipopolysaccharides. Serotonin can influence behavior by interacting with the vagus nerve and enteric neurons and acting on receptors in the gut. Some gut microbiota also produce serotonin directly.

Attraction is linked to reduced serotonin (40), a hormone involved in appetite and mood regulation (41). Moreover, a reduction in serotonin transporters is also observed in people with obsessive-compulsive disorder (42). Therefore, altered serotonin and its link to obsessive traits may contribute to the overpowering infatuation experienced in the early stages of love. Despite being primarily associated with the brain, approximately 95% of the body’s serotonin is produced by enterochromaffin cells of the gut (43). Gut microbiota play a key role in synthesizing and releasing gut-derived serotonin (44). For example, gut microbial metabolites such as SCFAs and lipopolysaccharides influence the serotonergic system (Fig. 2). However, it is not well understood how certain metabolites produced in the gut lumen can make their way to receptors on the intestinal cell surface, transversing not only distance but also getting through two layers of mucus.

SCFAs (primarily butyrate) in the gut lumen stimulate tryptophan hydroxylase-1 expression in enterochromaffin cells (45), which increases serotonin production by the enterochromaffin cells (46). This regulatory role of microbiota on serotonin production suggests a potential link between the gut microbiome and attraction involving neurological (including serotonergic) pathways associated with “reward” behaviors. While the majority of peripheral serotonin does not cross the blood-brain barrier (the selective, semi-permeable barrier that protects the brain by controlling which substances can pass from the bloodstream into the brain tissue), it can influence the brain’s serotonergic system indirectly. Increased serotonin in the gut modulates the vagus nerve, which transmits signals to the brainstem and limbic structures, including the raphe nuclei—the primary source of central serotonin. Through this gut-brain communication, microbiota-induced serotonin production may contribute to the regulation of mood, social bonding, and attraction by influencing central serotonergic pathways involved in reward processing and emotional regulation. Moreover, many bacterial species produce serotonin—for instance, bacteria in the Enterobacteriaceae family of the Proteobacteria phylum (47). Therefore, gut microbiota also have direct roles in serotonin production and, thus, may influence the biological pathways to attraction.

The gut microbiome is linked to dopaminergic neurotransmission (48). Gut microbiota influence the mesocorticolimbic circuit, which is involved in the reward pathway—the same phenomenon associated with attraction. The gut microbiome-dependent production of metabolites can also stimulate the activity of transient receptor potential vanilloid subfamily member 1, which is expressed in sensory neurons (4). These are densely expressed in spinal and cranial neurons. Through this gut-derived stimulation, microbiota can elevate dopamine levels in the brain’s ventral striatum, a structure associated with attraction in humans (4). This highlights a potential pathway for gut microbiota to influence attraction-associated emotions.

*Drosophila melanogaster* studies suggest that the gut microbiome can impact mating preferences. For instance, Sharon et al. conducted an experiment where a population of *D. melanogaster* was divided into two groups: one group was reared on a molasses-based medium, and the other on a starch-based medium (49). Subsequently, when the two groups were mixed, the flies raised on molasses preferred mating with other “molasses flies,” whereas the “starch flies” preferred to mate with other starch-reared individuals. This mating preference was closely linked to the gut microbiome. When the flies were treated with antibiotics, the mating preferences were absent, suggesting that the gut microbiome was responsible for driving these preferences. To support this, the researchers inoculated the flies with microbiota from the respective media, and the within-group mating preference was present again. The mechanism by which the gut microbiome influences mating behavior in *Drosophila* may involve alterations in steroid levels that have similar functions to sex steroids in vertebrates. These changes in hormone levels, in turn, significantly impact the flies’ mating decisions. Research that extends these findings to test the potential of the gut microbiome to affect vertebrate mating preferences is needed.

### Attachment-associated emotions

Attachment often takes precedence in long-term relationships. Attachment plays a role in friendships, parent-infant bonding, social connections, and various other long-term intimacies. In humans and other mammals, oxytocin and vasopressin are the key hormones involved in enhancing biological processes of attachment (50). Oxytocin is often called the “cuddle hormone” and is strongly associated with bond-forming (51). Indeed, microbiome-oxytocin-love associations have been proposed in the past (52, 53). Oxytocin is produced in the hypothalamus (although evidence suggests it is also produced by peripheral organs, including the gastrointestinal tract), which extends into the posterior pituitary and releases this hormone (e.g., during activities such as sex, breastfeeding, and childbirth) and vasopressin (54). Although these activities may seem unrelated, they all serve as precursors to bonding and thus are important for partnerships and familial bonds.

Vasopressin has a role in attachment, particularly in the context of forming and maintaining monogamous pair bonds. Often referred to as the “bonding hormone,” vasopressin is a neuropeptide that influences social behaviors, including attachment and mate choice (55). In males, vasopressin is associated with territorial and aggressive behaviors, often displayed during mate competition. It also affects the brain’s reward and pleasure centers, reinforcing the bond between mates (56). Studies of monogamous prairie voles (*Microtus ochrogaster*) have shown that higher levels of vasopressin receptors in certain brain nuclei are associated with stronger pair bonding and social attachment (57). In humans, vasopressin levels have been linked to partner bonding and relationship quality (58). Research suggests that individuals with specific genetic variations in vasopressin receptor genes may be more prone to forming strong and lasting attachments to their partners (59). Furthermore, vasopressin interacts with other neurotransmitters and hormones, including oxytocin and dopamine, to regulate social behaviors and emotional responses related to attachment and attraction (60). The interplay between these neurochemicals and their receptors in the brain is thought to shape the complex landscape of human social bonding and romantic relationships.

**Fig 3:**
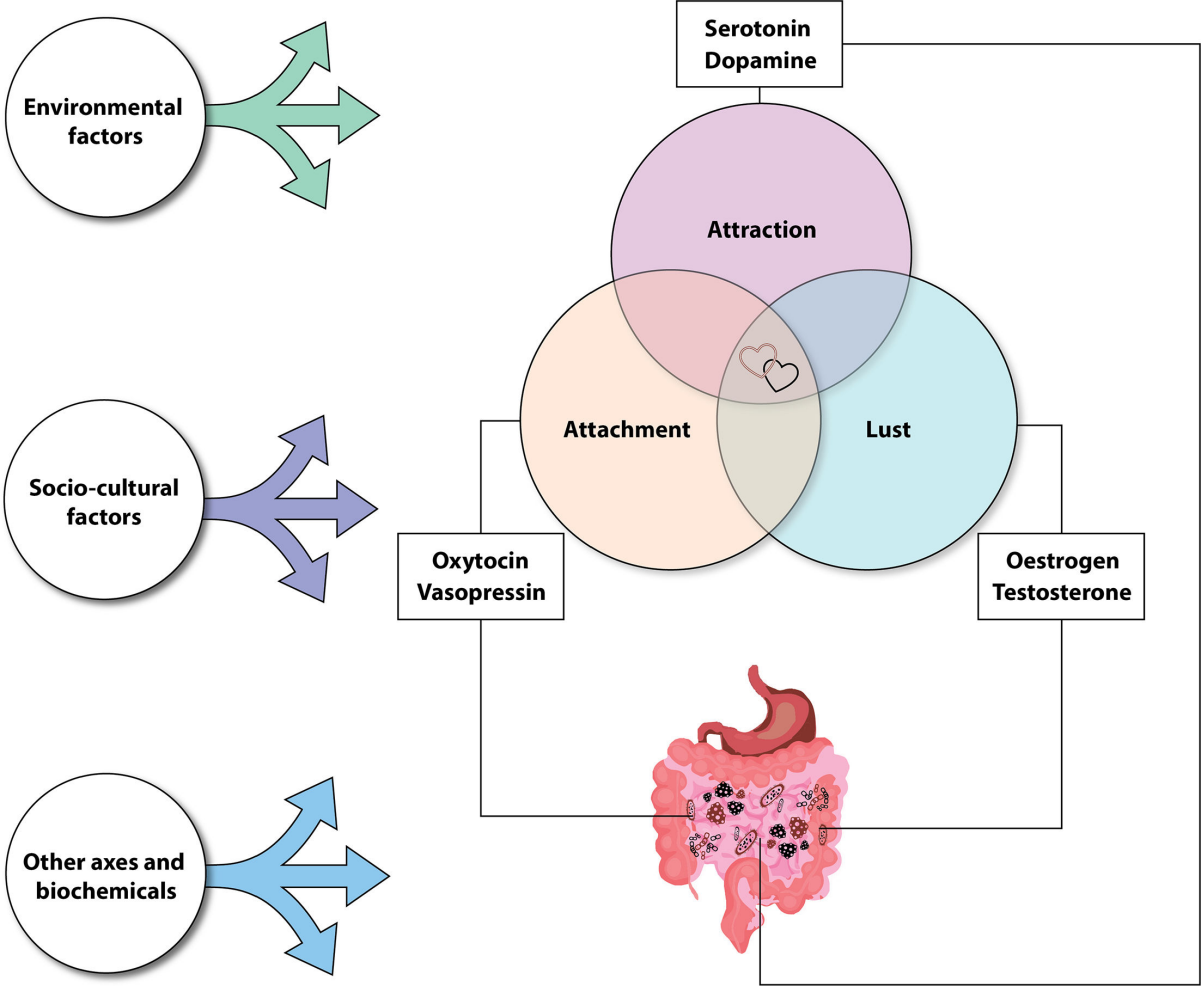
The potential role of microbiota on attachment-associated emotions (e.g., via oxytocinergic pathways) completes the core overlapping elements of our hypothesis. It is essential to acknowledge the complexity of other factors (e.g., environmental, socio-cultural, and other axes, such as the hypothalamic-pituitary-adrenal axis and cortisol) that influence human emotions and gut microbiomes.

The gut microbiome is involved in oxytocinergic signaling via the microbiota-gut-brain axis, contributing to the regulation of social behavior (61). Moreover, treating humans with a multispecies probiotic (3× *Bifidobacteria* and 3× *Lactobacilli*) increased endogenous oxytocin levels (62), which correlate with gut microbiome composition (e.g., *Roseburia*, *Parabacteroides*, Lachnospiraceae NK4A136 group, Ruminococcaceae UCG-013, and *Butyricicoccus*) (63). These microbes may contribute to oxytocinergic regulation via their production of SCFAs and other neuroactive metabolites. Therefore, the gut microbiome may influence the biological basis of attachment-associated emotions through its effects on oxytocin signaling (Fig. 3). However, further research is needed to elucidate the mechanisms by which microbial communities modulate central oxytocin release and social bonding behaviors.

Microbiota can also potentially influence vasopressin—the other key hormone involved in attachment-associated emotions. The live inoculation of *Limosilactobacillus reuteri* in rodents altered their vasopressin 1a-receptor in the paraventricular nucleus of the hypothalamus (64). Vasopressin 1a-receptor (also known as arginine vasopressin receptor 1a) is one of the three major receptor types for vasopressin and is present throughout the brain and peripheral organs (65). In mammals, arginine vasopressin is commonly associated with courtship and other social behaviors (66). This further highlights the potential role of microbiota in attachment-associated emotions.

### The holobiont blindspot, olfaction, and attraction

Animal host symbiotic microbiota can affect their decision-making via olfactory pathways. For instance, in a recent experiment with *Caenorhabditis elegans*, researchers showed that certain gut bacteria can imitate host sensory receptor molecules with the potential to influence the decisions of the host (67). This study focused on a commensal gut bacterium *Providencia* sp*.,* which produced the neuromodulator tyramine. This tyramine compound interacted with the host’s olfactory system, altering the host’s aversive responses to specific odors. This mechanism appears to promote mutually beneficial food decisions. Here, the gut bacterium manipulates the host animal so that a food source benefits both the animal host and the commensal bacteria. While microbiota-mediated feeding decisions might not directly relate to the attraction component of love, the olfactory system does—via the detection and processing of biochemical attractants (68).

These findings align with the holobiont theory, which conceptualizes the host and its associated microbiota as an integrated ecological unit subject to co-evolutionary processes (69). Rather than viewing the host organism as a standalone entity, the holobiont framework suggests that behavioral and physiological traits—including sensory processing and decision-making—may emerge from interactions between host and microbial genomes (70). In this context, microbial metabolites such as tyramine are potentially co-regulators of host behavior, shaped by evolutionary pressures that favor mutual benefit. This paradigm challenges traditional boundaries between “self” and “other” and suggests that what we often interpret as internally driven behavior may, in fact, be co-authored by our microbial symbionts.

Underappreciating the role that symbiotic microbiota have in influencing a host’s behavior was recently termed the “holobiont blindspot” (71). The authors recently proposed a thought experiment from an olfactory and attraction perspective in humans: (i) modifications or inter-individual differences in the human microbiome occur via environmental disturbances (e.g., via pollution exposures, changes in diet or antibiotic usage); (ii) this may lead to changes in odor perception in the human host; (iii) in turn, this may change host preferences (e.g., human odors as biochemical attractants); (iv) the question then becomes: could the host become less (or more) attracted to another individual due to a microbially mediated driver?; (v) in theory, this could have important relationship implications. This proposed pathway highlights the role that the human gut microbiome could play in complex social relationships because of changes in a host’s biology. In the future, it might be possible to design therapeutic interventions using microbial ecology knowledge (Box 1).

Box 1 **Microbial therapeutics and future directions**

Gaining insights into the potential microbial basis for love-associated emotions could be important for understanding—and repairing—relationship challenges. For instance, to what degree do human symbiotic microbiota associate with dysregulated love-associated hormone levels? Therapeutics could be in the form of a holistic intervention, such as changing diets, environmental exposures, or dietary supplements. Recent research has explored the possibility of altering gut microbiota to treat sexual dysfunction (72, 73). It is important to emphasize, though, that a lack of desire should not be invariably pathologized. Asexuality is a natural variation in the human experience, and treatments should only be given to those who desire them.

A prebiotic mixture can change the gut microbiome composition of rodents, increase estradiol (a potent oestrogen), and reduce sexual dysfunction (73). Research could conceivably augment the field of “psychobiotics,” which proposes that prebiotics and probiotics, when ingested in sufficient quantities, may confer a mental health benefit via the microbiota-gut-brain axis (74, 75). Indeed, the probiotic *Limosilactobacillus reuteri* can increase circulating oxytocin levels and its expression in the brain (61).

There are also environmental influences on the love-associated emotions. Environmental stimuli can influence hormone and neurotransmitter synthesis and secretion (76). For instance, people living in areas with more green spaces have higher dopamine levels (77) and lower circulating cortisol. As mentioned, hormones may shape the gut microbiome, and environmental stimuli could influence the endocrine-microbiota-love axis. Moreover, environments also provide microbial exposures (i.e., exposure to biodiverse aerobiomes could alter the human microbiome) (78, 79) that may contribute to hormone regulation and, thus, lust, attraction, and attachment. In the future, “green prescriptions” (80) (prescribed nature-based health interventions) might form part of relationship therapy. However, adverse environmental stimuli could affect our hormone levels and thus influence the endocrine-microbiota-love axis. For instance, foods and polluted air often contain high levels of endocrine disruptors (81), which might alter the endocrine function and the microbiome (82). Moreover, because quality environments and diets are not equally distributed and available, the environment-endocrine-microbiota-love axis could be viewed as a facet of social equity (83, 84).

## A BI-DIRECTIONAL RELATIONSHIP

While much of the focus in this article is on how gut microbiota can influence host endocrine signaling, it is important to recognize that this relationship is bidirectional. Hormones produced by the host—such as cortisol, sex steroids, and neuropeptides—can influence the composition, diversity, and activity of the gut microbiota (85). For example, elevated glucocorticoid levels associated with stress have been shown to alter gut microbial community structure (86). Similarly, sex hormones such as estrogens and androgens can shape microbial communities, potentially contributing to sex-based differences in microbiome profiles (87). These hormonally driven shifts in microbiota may in turn affect immune responses, metabolite production, and gut-brain communication, potentially creating feedback loops that influence mood, motivation, and emotional regulation. As Hokanson et al. said, *“*sex steroids shape the structure of the gut microbiota, and these microbes in turn regulate levels of bioactive sex steroids*”* (85). Incorporating this reciprocal dynamic will be essential for understanding the full complexity of microbiota-endocrine-emotion interactions.

To advance our understanding of microbiota-endocrine-emotion interactions, there is a clear need for integrative studies that simultaneously capture human psychological data alongside microbiome composition and endocrine biomarkers. Such multidisciplinary approaches will be critical for uncovering causal links, identifying feedback loops, and distinguishing correlation from mechanistic influence in the complex, dynamic interplay between microbes, hormones, and emotion-related behaviors.

## CONCLUDING REMARKS

Our gut microbiome may affect love-associated emotions through effects on hormonal mechanisms. Acknowledging the multifaceted dimensions of human existence, including diversity in gender identity and sexual orientation, we explored the evidence that love-associated emotions (often denoted, perhaps too simplistically, as the heuristic categories of “lust,” “attraction,” and “attachment”) are influenced biologically by a suite of hormones. These include testosterone and estradiol, serotonin and dopamine, and oxytocin and vasopressin. Importantly, the human microbiome and its metabolites can interact with endocrine system pathways and influence the production of these hormones, thus providing a potential microbiological basis for love-associated emotions. This hypothesis could have important implications for understanding relationships. It could also prompt us to consider the role of microbiomes in contrasting emotions and behaviors, like hate and aggression. Future research on this topic, including the complex environmental factors that influence our emotions and microbiomes, is needed.

## AFTER EPUB

*[This article was published on 14 July 2025 with missing text and references. The text and References were corrected in the current version, posted on 16 July 2025.]*
